# Association Between Dietary Fiber and Parkinson's Disease in the United States: NHANES 2005–2018

**DOI:** 10.1002/brb3.70819

**Published:** 2025-09-16

**Authors:** Qifan Yang, Shijin Li, Xiao Li, Jiayu Peng, Bowen Han, Yuetong Liu, Xuehui Chang

**Affiliations:** ^1^ Henan University of Chinese Medicine Zhengzhou Henan China; ^2^ Zhengzhou Seventh People's Hospital Zhengzhou Henan China; ^3^ Yuzhou People's Hospital Yuzhou Henan China

**Keywords:** cross‐sectional study, dietary fiber, dietary therapy, National Health and Nutrition Examination Survey (NHANES), Parkinson's disease

## Abstract

**Objective:**

To explore the association between dietary fiber (DF) intake and the prevalence of Parkinson's disease (PD) in American adults on the basis of cross‐sectional data.

**Methods:**

This cross‐sectional study utilized data from the National Health and Nutrition Examination Survey (NHANES) spanning from 2005 to 2018. Multivariable logistic regression models and trend analyses were employed to examine the relationship between average dietary fiber intake (ADFI, g/day) and PD prevalence. Additionally, restricted cubic spline (RCS) models were used to investigate the potential nonlinear dose–response relationship between ADFI and PD. Subgroup interaction analyses were conducted as exploratory analyses to evaluate the association between ADFI and PD across different subgroups.

**Results:**

A total of 30,884 adults aged 20 years and older were included in the study, among whom 411 were PD patients. Adjusted multivariable logistic regression models (low vs. high ADFI: odds ratio [OR]: 1.68, 95% confidence interval [CI]: 1.15–2.46, *p* = 0.008) and trend analyses (OR: 0.84, 95% CI: 0.74–0.95, *p* = 0.007) indicated a significant inverse association between ADFI and PD prevalence. The RCS regression analysis demonstrated a significant linear relationship between ADFI and PD (*p* for overall = 0.002, *p* for nonlinearity = 0.213). Subgroup interaction analysis revealed that the association between higher ADFI and lower PD risk was more pronounced in females and non‐stroke individuals.

**Conclusion:**

Higher ADFI was associated with lower odds of PD in this cross‐sectional study. Specifically, low ADFI was associated with 68% higher odds of PD, whereas each unit increase in ADFI was associated with 16% lower odds of PD.

## Graphical abstract

Higher dietary fiber intake is associated with lower odds of Parkinson's disease in US adults (NHANES 2005–2018), with low intake linked to 68% higher odds and each unit increase tied to 16% lower odds.

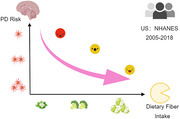

## 1 Introduction

Parkinson's disease (PD), also known as paralysis agitans, is a common neurodegenerative disorder in middle‐aged and elderly individuals. Patients exhibit motor symptoms such as resting tremor and bradykinesia, as well as nonmotor symptoms such as constipation, olfactory dysfunction, and sleep disturbances. According to the Global Burden of Disease Study 2021, the global burden of PD has increased from 2.5 million in the 1990s to 11.76 million, causing approximately 388,000 deaths in 2021. With the aging population and increasing life expectancy, this trend is expected to continue. In the United States, it is projected that by 2030, there will be approximately 1,238,000 individuals with PD (Marras et al. 2018; Ben‐Shlomo et al. 2024; GBD 2021 Causes of Death Collaborators 2024; GBD 2021 Diseases and Injuries Collaborators 2024). The pathological features of PD include the progressive degeneration of dopaminergic neurons in the substantia nigra and the formation of Lewy bodies. Although the exact pathogenesis of PD remains unclear, research suggests that factors such as gut microbiota imbalance and oxidative stress are closely associated with the onset of PD (Sampson et al. 2016; Raza et al. 2019).

Dietary fiber (DF) typically consists of polysaccharide forms from plant‐based foods, which are carbohydrates that cannot be digested in the human gut. These compounds include non‐starch polysaccharides, cellulose, pectins, hydrocolloids, fructo‐oligosaccharides, and resistant starch (Hijová et al. 2019). DF can regulate the gut microbiota, and the phenolic compounds contained within it can produce antioxidant effects (Angulo‐López et al. 2022; Um et al. 2023). DF intake is an integral part of human life and is closely related to human health and the pathology of various diseases. Consuming foods rich in DF can effectively reduce the risk of metabolic, cardiovascular, autoimmune, and neurological diseases. Conversely, a lack of DF can alter the gut microbiota–hippocampus axis, leading to cognitive impairment and negatively affecting brain function. Excessive DF intake, however, may lead to risks, such as bloating, gastrointestinal gas, and even intestinal obstruction (Barber et al. 2020; Shi et al. 2021; Ioniță‐Mîndrican et al. 2022). Notably, the recommended DF intake for European and American populations is 30–50 g/day for men and 25–32 g/day for women. However, the actual DF intake varies across different countries and regions. Surveys indicate that the actual intake for European adults is 18–24 g/day for men and 16–20 g/day for women. On the basis of data from the National Health and Nutrition Examination Survey (NHANES), the average DF intake in North American populations is even lower than that in European populations (Stephen et al. 2017).

Current research has focused primarily on dietary management and the interaction between food and medication in populations already diagnosed with PD, demonstrating that DF intake can have a positive effect on PD patients (Baert et al. 2020; Agnieszka et al. 2022). However, studies exploring the association between DF intake levels and PD prevalence are relatively rare. It is necessary to further investigate the relationship between DF consumption and PD. Therefore, this study aimed to investigate the potential association between average dietary fiber intake (ADFI) and PD, which could have significant implications for the prevention and treatment of this disease.

## 2 Methods

### 2.1 Data Source and Study Population

**FIGURE 1:**
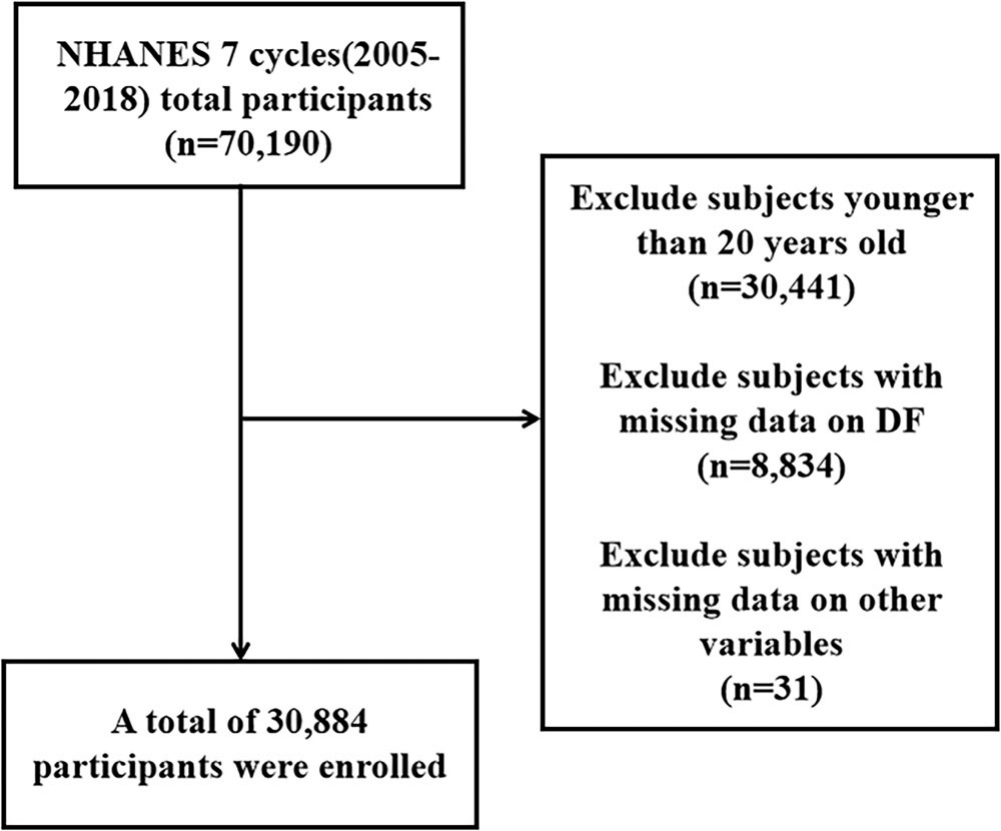
Flowchart of participant selection. DF, dietary fiber.

This study leverages data from the NHANES website spanning from the years 2005 to 2018. NHANES is a large, nationally representative cross‐sectional study of the US population conducted every 2 years via a multistage stratified sampling design. It is administered by the Centers for Disease Control and Prevention (CDC) to assess the health and nutritional status of noninstitutionalized US civilians (detailed data information and sampling design methodology are available on the NHANES official website: https://www.cdc.gov/nchs/nhanes/index.htm). In this study, we initially selected 70,190 subjects from seven consecutive NHANES survey cycles from 2005 to 2018. To ensure consistency in age baseline and considering that PD typically does not occur in minors (Ben‐Shlomo et al. 2024), we excluded 30,441 participants under the age of 20. Additionally, we excluded 8834 participants who lacked data on DF intake for any given day and 31 participants with missing information on other variables. This resulted in a final study population of 30,884 individuals. The recruitment process is depicted in Figure 1.

### 2.2 Measurement of DF

DF intake was assessed at the mobile examination center (MEC) for participants across all age groups. The MEC dietary interview details are provided in the NHANES Dietary Database Protocol and Procedure and Measuring Guides for the Dietary Recall Interview, which aims to estimate the total intake of food energy (calories), nutrients, and non‐nutrient food components from foods and beverages consumed in the 24 h prior to the interview (from midnight to midnight). For quality control, NHANES utilizes the automated multiple‐pass method (AMPM) from the USDA for dietary data collection. The dietary intake data are processed via the USDA's Food and Nutrient Database for Dietary Studies (FNDDS), a reliable data source for US government food fortification policies and child nutrition policies. This approach ensures a high level of accuracy and adequacy in recording dietary intake, including DF. In this study, ADFI (g/day) was calculated as follows: ADFI (g/day) = [DF intake on Day 1 (g) + DF intake on Day 2 (g)]/2. On the basis of the distribution range of ADFI, participants were divided into four quartiles (Q1–Q4) via the interquartile range method. The quartiles are defined as follows: Q1 (<10.85 g/day), Q2 (≥10.85 and <15.40 g/day), Q3 (≥15.40 and <21.30 g/day), and Q4 (≥21.30 g/day).

### 2.3 Diagnosis of PD

To identify whether participants had PD, we utilized responses to prescription medication‐related questions in NHANES. Participants were classified as having PD based on their reported use of one or more medications specifically prescribed for the treatment of PD. The types of PD prescription medications were identified via the drug records and codes available in NHANES. Individuals actively treated with PD medications were classified as having PD, whereas participants who did not report the use of identified anti‐PD medications were classified as not having PD (DeMarco et al. 2021). Although this method has certain limitations, such as potential underreporting or misclassification of medication use, it currently represents the best available method for classifying the PD status of study participants.

### 2.4 Measurements of Other Covariates

Demographic covariates, including age, sex, race, education level, and marital status, were derived from NHANES sociodemographic data. Age was categorized into four groups: 20–39, 40–59, 60–79, and 80 years or older. Energy intake covariates were obtained from the same source as DF and include total sugar (TSUG) and total fat (TFAT). The average daily intake of TSUG and TFAT was calculated using the following formulas: TSUG (g/day) = [SUG intake on Day 1 (g) + SUG intake on Day 2 (g)]/2, TFAT (g/day) = [FAT intake on Day 1 (g) + FAT intake on Day 2 (g)]/2. Comorbid conditions include stroke, diabetes, and hypertension, with information sourced from questionnaires, physical examinations, and laboratory data within the NHANES. The definition of stroke is determined on the basis of participants’ responses to the question “Ever told you had a stroke” in the medical conditions section. The prevalence of diabetes is assessed via multiple criteria. Primarily, it considers self‐reports in response to the diabetes‐related question “Doctor told you have diabetes.” Additionally, it incorporates laboratory findings where glycated hemoglobin (HbA1c) levels above 6.5% or fasting blood glucose (FBG) levels exceeding 126 mg/dL are considered according to the American Diabetes Association's definition. Hypertension status is also defined via multiple standards. It primarily references self‐reports in response to the hypertension‐related questions “Ever told you had high blood pressure?” and “Now taking prescribed medicine for HBP.” Furthermore, it integrates examination data by analyzing the mean of four blood pressure readings, considering an average systolic blood pressure above 140 mmHg and an average diastolic blood pressure above 90 mmHg.

### 2.5 Statistical Analyses

Data processing and analysis were conducted using the open‐source statistical software R (version 4.4.1), along with Zstats v1.0 (www.zstats.net), to perform weighted analyses on the included sample data. All statistical analyses were weighted as described in the NHANES manual to create a nationally representative sample. Continuous variables are presented as weighted means ± standard error, whereas categorical variables are expressed as observed frequencies and weighted percentages.

First, a basic statistical description of the baseline characteristics of the participants was conducted. Second, to analyze the association between DF and PD, multiple logistic regression analysis was employed to calculate the odds ratios (ORs) and 95% confidence intervals (CIs), and four covariate models were evaluated: Model 1 with no adjustments. Model 2 was adjusted for age (20–39, 40–59, 60–79, and ≥80 years), sex, race, marital status, education level, smoking status, and alcohol consumption as categorical variables. Model 3 added comorbid conditions of stroke, diabetes, and hypertension to the adjustments in Model 2. Model 4 further included TSUG and TFAT energy intake covariates in addition to Model 3, with trend analysis conducted across all four models (using the median ADFI as the node value). To recognize the potential adverse effects of both inadequate and excessive DF intake, we subsequently employed restricted cubic spline (RCS) regression to assess potential nonlinear and dose–response relationships between DF and PD. Finally, subgroup interaction analysis via NHANES‐weighted methodology was performed to explore the variations in the association between ADFI and PD across different subgroups. A *p* value < 0.05 (two‐tailed) was considered statistically significant.

## 3 Results

### 3.1 General Characteristics of the Study Population

Table 1 displays the baseline characteristics of 30,884 participants from a cross‐sectional survey conducted between 2005 and 2018, categorized by quartiles of ADFI. Among these participants, 411 individuals (1.27%) were diagnosed with PD. The weighted mean age of all participants was 47.95 years, with 47.31% being male and 52.69% being female. Baseline characteristics were analyzed on the basis of ADFI quartiles. As the quartiles of ADFI increased, participants tended to be predominantly middle‐aged (*p* < 0.001), and the proportion of males increased (*p* < 0.001). There was a rise in the proportion of Mexican American participants and a decrease in the proportion of non‐Hispanic Black participants (*p* < 0.001). Additionally, the number of married participants increased (*p* < 0.001), as did the proportion of individuals with a college education or higher (*p* < 0.001). Furthermore, the proportion of nonsmokers increased (*p* < 0.001), and the proportion of drinkers also increased (*p* < 0.001). The prevalence of stroke and hypertension decreased (*p* < 0.001), as did the prevalence of diabetes (*p* = 0.015). Both TSUG and TFAT showed a gradually increased (*p* < 0.001). Moreover, the baseline table indicates that as ADFI increased, the incidence of PD gradually decreased (*p* = 0.003), with the prevalence of PD decreasing from 1.66% in Q1 to 0.90% in Q4. Detailed data can be found in Table S1.

**TABLE 1:** The characteristics of participants.

	Total	Q1	Q2	Q3	Q4	*p* value
Number	30,884	8292	7748	7317	7527	
DF(mean [SD])	16.98 ± 0.12					
Age (years) (%)	47.95 ± 0.23					<0.001
20–39	10,134 (34.92)	2817 (37.48)	2589 (35.36)	2299 (32.45)	2429 (34.38)	
40–59	10,193 (38.05)	2708 (37.34)	2413 (35.85)	2405 (39.23)	2667 (39.76)	
60–79	8630 (22.78)	2237 (20.58)	2192 (23.84)	2108 (23.78)	2093 (22.91)	
≥80	1927 (4.25)	530 (4.60)	554 (4.95)	505 (4.53)	338 (2.95)	
Gender (%)						<0.001
Female	16,188 (52.69)	5051 (62.78)	4427 (57.54)	3727 (51.45)	2983 (39.12)	
Male	14,696 (47.31)	3241 (37.22)	3321 (42.46)	3590 (48.55)	4544 (60.88)	
Race (%)						<0.001
Mexican American	4660 (7.92)	786 (5.23)	995 (6.66)	1155 (7.81)	1724 (11.93)	
Non‐Hispanic Black	6707 (11.02)	2509 (16.69)	1863 (12.46)	1346 (8.73)	989 (6.24)	
Non‐Hispanic White	13,584 (68.91)	3563 (65.94)	3481 (69.31)	3379 (71.88)	3161 (68.51)	
Other	5933 (12.16)	1434 (12.14)	1409 (11.57)	1437 (11.59)	1653 (13.32)	
Marital (%)						<0.001
Yes	18,660 (64.57)	4394 (57.22)	4532 (63.11)	4638 (67.59)	5096 (70.29)	
No	12,224 (35.43)	3898 (42.78)	3216 (36.89)	2679 (32.41)	2431 (29.71)	
Education (%)						<0.001
Less than High School	7190 (14.92)	2324 (19.87)	1670 (13.97)	1535 (13.18)	1661 (12.71)	
High School Graduate	7115 (23.22)	2255 (29.06)	1963 (26.26)	1595 (21.71)	1302 (15.93)	
College Graduate or above	16,579 (61.85)	3713 (51.08)	4115 (59.78)	4187 (65.11)	4564 (71.36)	
Smoke (%)						<0.001
Yes	13,769 (44.52)	4286 (52.40)	3397 (44.66)	3066 (41.32)	3020 (39.73)	
No	17,115 (55.48)	4006 (47.60)	4351 (55.34)	4251 (58.68)	4507 (60.27)	
Alcohol (%)						<0.001
Yes	8930 (33.59)	2265 (30.72)	2170 (32.93)	2176 (34.81)	2319 (35.88)	
No	21,954 (66.41)	6027 (69.28)	5578 (67.07)	5141 (65.19)	5208 (64.12)	
Stroke (%)						<0.001
Yes	1215 (2.92)	468 (4.19)	325 (3.41)	245 (2.29)	177 (1.78)	
No	29,669 (97.08)	7824 (95.81)	7423 (96.59)	7072 (97.71)	7350 (98.22)	
Diabetes (%)						0.015
Yes	5420 (13.05)	1528 (13.23)	1442 (14.31)	1261 (12.83)	1189 (11.87)	
No	25,464 (86.95)	6764 (86.77)	6306 (85.69)	6056 (87.17)	6338 (88.13)	
Hypertension (%)						<0.001
Yes	13,312 (38.12)	3850 (40.08)	3448 (39.54)	3097 (37.61)	2917 (35.28)	
No	17,572 (61.88)	4442 (59.92)	4300 (60.46)	4220 (62.39)	4610 (64.72)	
Parkinson (%)						0.003
Yes	411 (1.27)	128 (1.66)	121 (1.40)	92 (1.11)	70 (0.90)	
No	30,473 (98.73)	8164 (98.34)	7627 (98.60)	7225 (98.89)	7457 (99.10)	
						
TSUG (mean [SD])	109.94 (64.53)	87.58 (59.05)	102.00 (59.12)	115.28 (60.35)	134.69 (69.43)	<0.001
TFAT (mean [SD])	80.87 (38.16)	59.02 (26.24)	75.61 (30.51)	86.25 (35.11)	102.40 (44.35)	<0.001

*Note*: Continuous variables are presented as weighted mean ± standard error (SE); categorical variables are presented as counts (weighted percentage); *p* values are weighted.

Abbreviation: DF, dietary fiber.

### 3.2 Multivariable Logistic Regression Analysis of the Association Between DF and PD

The relationship between ADFI and PD among American adults was analyzed via four weighted logistic regression models, as shown in Table 2. Model 1 had no covariate adjustments; Model 2 adjusted for age, sex, race, marital status, education level, smoking status, and alcohol consumption; Model 3 added comorbid conditions such as stroke, diabetes, and hypertension; and Model 4 included complete dietary energy intake adjustments with TSUG and TFAT. In all models, especially after adjusting for all covariates, we found that individuals in the lower quartiles of ADFI had a significantly greater risk of PD than those in the higher quartiles (Model 4, OR: 1.68, 95% CI: 1.15–2.46, *p* = 0.008). Detailed data can be found in Table S2.

**TABLE 2:** Association between quartiles of average dietary fiber intake (ADFI) and Parkinson's disease (PD).

		Model 1	Model 2	Model 3	Model 4
Q1	OR (95% CI)	1.86 (1.36, 2.53)***	1.50 (1.09, 2.06)*	1.45 (1.05, 2.00)*	1.68 (1.15, 2.46)**
Q2	OR (95% CI)	1.56 (1.12, 2.19)*	1.33 (0.95, 1.87)	1.29 (0.91, 1.82)	1.44 (0.99, 2.10)
Q3	OR (95% CI)	1.24 (0.87, 1.75)	1.09 (0.76, 1.56)	1.08 (0.76, 1.55)	1.17 (0.82, 1.68)
Q4	OR (95% CI)	Ref	Ref	Ref	Ref
Trend analysis	OR (95% CI)	0.81 (0.74, 0.90)***	0.87 (0.78, 0.96)**	0.88 (0.79, 0.97)*	0.84 (0.74, 0.95)**
*p* for trend		<0.001	0.006	0.015	0.007

*Note*: Model 1: Unadjusted. Model 2: Adjusted for age, gender, race, married/live with partner status, education level, smoking status, and alcohol use. Model 3: Model 2 plus additional adjustment for disease histories, including stroke, diabetes, and hypertension. Model 4: Model 3 plus additional adjustment for TSUG and TFAT dietary energy intake.

Abbreviations: CI, confidence interval; OR, odds ratios.

***p < 0.001, **p < 0.01, and *p < 0.05.

In the trend analysis, the median ADFI value was used as the node value. The results revealed that the ORs in all models were less than 1, indicating a reverse association between increased ADFI and the risk of PD, as shown in Table 2. Although the OR values varied slightly among the models, the overall trend was consistent, particularly in Model 4, where an increase in ADFI was associated with a decreased risk of PD (Model 4, OR: 0.84, 95% CI: 0.74–0.95, *p* = 0.007). Detailed data can be found in Table S3.

### 3.3 Linear Associations Between DF and the Risk of PD

**FIGURE 2:**
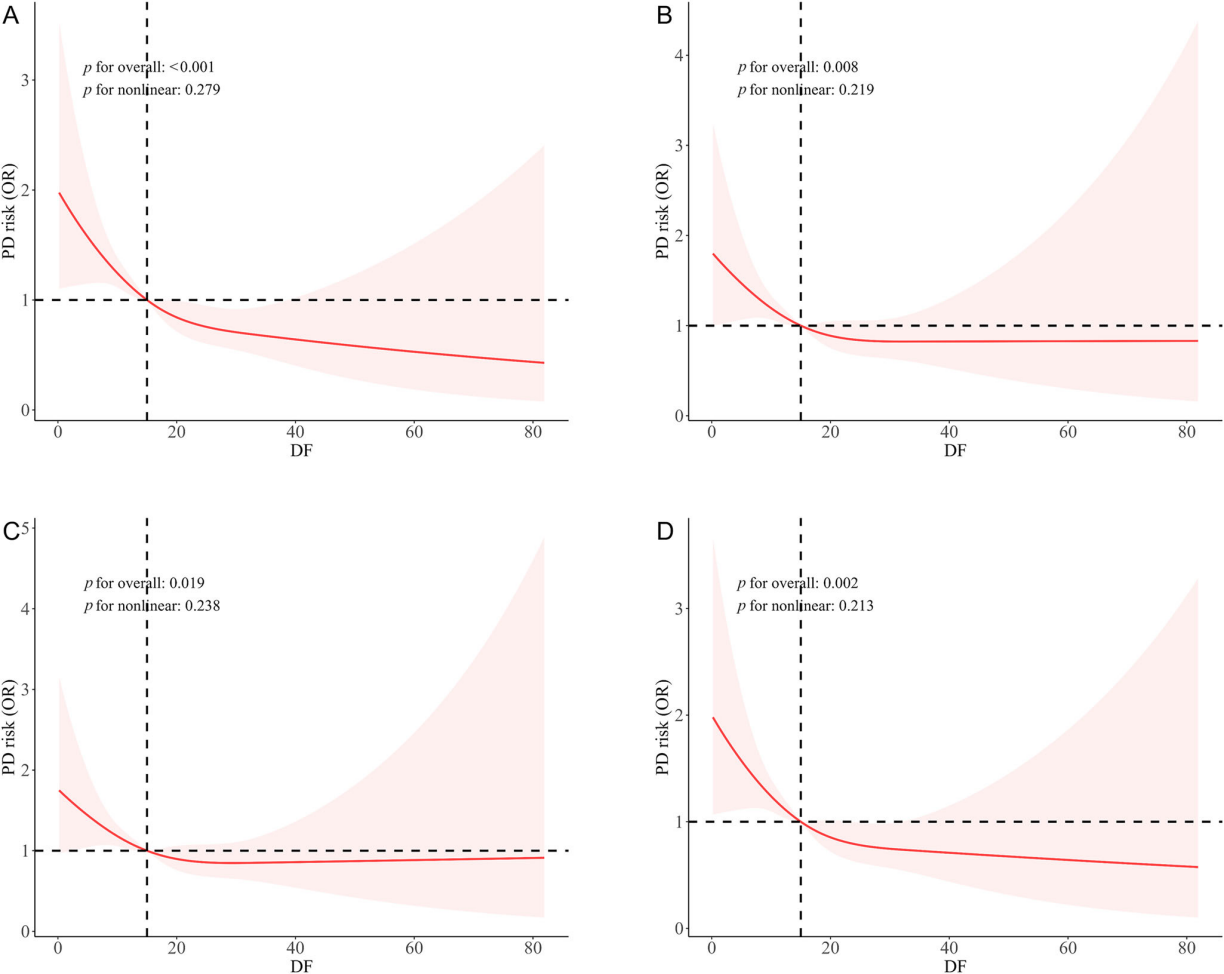
Restricted cubic spline (RCS) analysis of the association between ADFI and PD. The *x*‐axis represents the ADFI level (g/day), and the *y*‐axis represents the relative probability of developing PD. (A) Association between ADFI level and PD without adjustment. (B) Association between ADFI level and PD adjusted for age, sex, race, marital status, education level, smoking, and drinking habits. (C) Association between ADFI level and PD with additional adjustment for stroke, diabetes, and hypertension. (D) Association between ADFI and PD with further adjustment for dietary energy intake (TSUG and TFAT). OR, odds ratio; PD, Parkinson's disease.

This study employed RCS plots to visually illustrate the dose–response relationship between ADFI and PD and to explore the potential nonlinear relationship. Additionally, four models were developed to adjust for different confounding factors. The results of all four RCS models indicate a significant association between higher ADFI and lower PD prevalence odds, without evidence of nonlinearity. The RCS plots of the four models are shown in Figure 2. Detailed data can be found in Table S4.

### 3.4 Subgroup Interaction Analysis of the Association Between DF and PD

To further explore any specific subgroups within the survey, we conducted a subgroup analysis via NHANES weighted methods. As shown in Table 3, we divided the population into multiple subgroups based on age, sex, race, marital status, education level, smoking and alcohol consumption, and comorbid conditions of stroke, diabetes, and hypertension, as well as energy intake stratified by the quartiles of TSUG and TFAT. Our results indicate that, except for sex and stroke status, the interaction results for other subgroups were consistent, with no statistically significant interactions observed.

**TABLE 3:** Subgroup interaction analysis of the association between average dietary fiber intake (ADFI) and Parkinson's disease (PD).

	OR (95% CI)	*p* value	*p* for interaction
Age			0.509
20–39	0.99 (0.95, 1.04)	0.737	
40–59	0.97 (0.94, 0.99)	0.022	
60–79	0.97 (0.95, 0.99)	0.049	
≥=80	0.95 (0.91, 0.99)	0.018	
Gender			0.038
Female	0.95 (0.93, 0.98)	0.001	
Male	0.99 (0.97, 1.01)	0.371	
Race			0.853
Mexican American	0.99 (0.93, 1.04)	0.621	
Non‐Hispanic Black	0.99 (0.95, 1.03)	0.594	
Non‐Hispanic White	0.97 (0.95, 0.99)	0.009	
Other	0.97 (0.91, 1.03)	0.284	
Marital			0.687
Yes	0.97 (0.95, 0.99)	0.002	
No	0.98 (0.95, 1.01)	0.129	
Education			0.433
Less than high school	0.99 (0.96, 1.02)	0.576	
High school graduate	0.98 (0.94, 1.02)	0.279	
College graduate or above	0.96 (0.94, 0.99)	0.010	
Smoke			0.091
Yes	0.98 (0.96, 1.01)	0.219	
No	0.96 (0.93, 0.98)	0.001	
Alcohol			0.283
Yes	0.96 (0.92, 0.99)	0.019	
No	0.98 (0.96, 1.00)	0.026	
Stroke			0.005
Yes	1.03 (1.00, 1.07)	0.089	
No	0.97 (0.95, 0.99)	0.002	
Diabetes			0.091
Yes	1.00 (0.97, 1.02)	0.723	
No	0.97 (0.95, 0.99)	0.003	
Hypertension			0.114
Yes	0.99 (0.97, 1.01)	0.174	
No	0.95 (0.92, 0.99)	0.015	
TSUG			0.800
0–65.180	0.97 (0.93, 1.02)	0.233	
65.181–97.280	0.95 (0.91, 1.00)	0.046	
97.281–139.435	0.97 (0.94, 1.01)	0.113	
≥=139.436	0.98 (0.95, 1.01)	0.136	
TFAT			0.227
0–54.185	0.95 (0.91, 0.98)	0.004	
54.186–74.505	0.96 (0.93, 1.00)	0.029	
74.506–100.715	0.99 (0.94, 1.03)	0.551	
≥=100.716	0.99 (0.96, 1.02)	0.532	

Abbreviations: ORs, odds ratios; CI, confidence interval.

The relationship between ADFI and PD was significantly different in the subgroups stratified by sex and stroke status, indicating that the association between increased ADFI and reduced risk of PD was more pronounced in the female subgroup (*p* for interaction = 0.038, OR: 0.95, 95% CI: 0.93–0.98, *p* = 0.001) and the non‐stroke subgroup (*p* for interaction = 0.005, OR: 0.97, 95% CI: 0.95–0.99, *p* = 0.002). Detailed data can be found in Table S5.

## 4 Discussion

This study utilized data from seven cycles of NHANES (2005–2018) to investigate the relationship between ADFI and PD. Our findings indicate that, after adjusting for other covariates, there is an inverse association between ADFI levels and PD risk. Individuals with low ADFI levels had 68% higher odds of PD prevalence compared to those with higher levels. Furthermore, higher ADFI (per unit) was associated with 16% lower odds of PD. Subgroup interaction analysis results revealed consistent interaction results across all subgroups, except for sex and stroke status, where significant interactions were observed. Therefore, the potential mechanisms underlying the relationship between ADFI and PD in female and non‐stroke subgroups warrant further in‐depth research and exploration.

DF is an essential nutrient for human health and is widely found in plant‐based foods, such as whole grains, vegetables, and fruits. DF is primarily categorized into two types: soluble and insoluble. Soluble DF can dissolve in water to form a gel‐like substance, which is derived mainly from oats, legumes, apples, and citrus fruits. This type of fiber helps lower blood glucose levels and cholesterol, promotes gut health, and may play a positive role in the prevention of cardiovascular diseases (Zhang et al. 2022). In contrast, insoluble DF does not dissolve in water; it increases stool bulk and promotes intestinal peristalsis, with primary sources including whole grains, nuts, and vegetables. This type of fiber aids in preventing constipation and maintaining gut health (Korczak and Slavin 2020). Given the positive impact of a DF‐rich diet on human health, interest in the relationship between DF and neurological health, particularly in relation to PD, has increased in recent years (Gallop et al. 2021).

A study investigating the relationships among diet, microbiota, and the onset of PD revealed that a Mediterranean diet, which is rich in a variety of plant foods, can reduce the risk of PD (Jackson et al. 2019). Similarly, a prospective study based on the UK Biobank indicated that incorporating readily available vegetables into a habitual diet is associated with a lower risk of PD (Tresserra‐Rimbau et al. 2023). Moreover, in individuals with PD, those who primarily consume plant‐based foods, compared with those with an omnivorous diet, can manage disease progression by improving their motor symptoms through a plant‐based diet (Baroni et al. 2011). Plant‐based foods are typically rich in DF. These studies highlight that, whether through adherence to a Mediterranean diet or a diet primarily consisting of plant‐based foods, increasing the proportion of DF in the diet is generally associated with a lower risk of PD.

However, contemporary dietary patterns are characterized by high consumption of ultra‐processed foods and a prevailing low intake of DF. This low‐DF dietary pattern may influence the composition and function of the gut microbiota, thereby potentially impacting the risk of PD (Martin‐Gallausiaux et al. 2021; Barbara et al. 2021; Brown 2019; Lively and Schlichter 2018). Research indicates that the gut microbiome profile in PD patients often differs significantly from that of healthy individuals, with particular impairments observed in functions related to short‐chain fatty acid (SCFA) production (Abreu Y Abreu et al. 2021). Studies have indicated that higher DF intake is associated with a reduced risk of several neurological disorders, including PD (Martínez Leo and Segura Campos 2020; Ramírez‐Salazar et al. 2021). The underlying mechanisms are likely linked to the fermentation of DF by gut microbes, which generates SCFAs. These SCFAs not only serve as an energy source for the gut epithelium but also possess anti‐inflammatory properties and exhibit the potential to improve gut barrier function (Tian et al. 2023). Furthermore, DF intake contributes to promoting the growth of beneficial bacteria (Donadio and Fabi 2024). These findings may inform the design of future prospective studies on DF interventions for PD.

Furthermore, neuroinflammation plays a critical role in the pathogenesis of PD. Evidence suggests that gut inflammation may influence neurological health through the gut–brain axis (Rolli‐Derkinderen et al. 2020; Huang et al. 2021). Studies have shown an inverse association between DF intake and systemic inflammation levels (Qi et al. 2023; Wu et al. 2024). Mechanistically, SCFAs derived from microbial fermentation of DF are proposed to exert anti‐inflammatory effects via G protein‐coupled receptor (GPCR) signaling pathways (Han et al. 2023). This may constitute one of the key biological pathways underlying the inverse relationship between DF intake and PD risk.

Regarding subgroup interaction analyses, the inverse association between increased ADFI intake and reduced PD risk was more pronounced in females. This gender disparity may be attributed not only to sex differences in nigrostriatal circuitry and potential neuroprotective effects of estrogen (Cerri et al. 2019; Gillies and McArthur 2010), but also to established dietary patterns: with advancing age, women demonstrate greater awareness in caloric control and protein selection, showing higher willingness to consume beneficial high‐DF foods (e.g., fruits and vegetables), whereas men tend to maintain consistent caloric/nutrient intake with greater consumption of less healthy food groups (Ahn et al. 2021; Feraco et al. 2024). Similarly, a stronger ADFI‐PD inverse association was observed in the stroke‐free subgroup. This aligns with consistent evidence that higher DF intake constitutes a protective factor against stroke, particularly ischemic stroke (Jin et al. 2024; Guo et al. 2022). Consequently, stroke‐free individuals typically represent populations characterized by healthier overall lifestyles, where elevated DF consumption serves as an integral component that may partly account for the enhanced association magnitude in this subgroup.

The RCS analysis revealed no significant nonlinear relationship between ADFI and PD (*p* for overall = 0.002, *p* for nonlinearity = 0.213), supporting a predominantly linear association where higher ADFI correlated with lower PD prevalence odds. Therefore, more studies are needed to understand how DF directly influences the onset and progression of PD. As previously mentioned, DF is classified into insoluble and soluble fibers. In addition to regulating gastrointestinal function, DF can maintain blood glucose levels and bind bile acids to reduce low‐density lipoprotein (LDL) cholesterol, thereby protecting cardiovascular health. To gain deeper insights, methods such as mass spectrometry can be utilized to explore the various bioactive components of DF fully. A multidisciplinary approach involving molecular cell experiments, in vivo animal studies, human randomized controlled trials, epidemiological surveys, human tissue research, and genetic studies should be employed to investigate the mechanisms of action of these bioactive components. We believe that our findings will encourage researchers in the PD field and those focusing on dietary modulation of neurological diseases to conduct more studies, potentially leading to improved PD prevention and treatment strategies. Future research should focus on collecting more detailed and representative data on daily dietary component intake and supplementing data on the direct impact of ADFI on PD pathogenesis. In addition, genome‐wide association studies (GWAS) should be conducted to identify genetic factors affecting DF digestion and utilization levels and their interaction with PD risk. Large‐scale longitudinal cohort studies should also be carried out to monitor ADFI levels and PD incidence over time.

This study has several limitations that need to be acknowledged. First, because the analysis was based on data from the NHANES database, the cross‐sectional design cannot establish causality. Therefore, future prospective cohort studies are needed to further clarify the impact of DF intake on patients with PD. Second, although key covariates were adjusted for in our models, the potential for residual confounding remains a significant limitation. Specifically, several important confounders not captured in the NHANES database—or not controlled for—could not be fully incorporated into our analysis. These include detailed measures of physical activity (intensity and duration), diverse dietary supplement usage, genetic susceptibility (e.g., family history of PD), and granular environmental toxin exposure data (such as pesticides, heavy metals, plastic‐related chemicals, radiation, and electromagnetic fields). The absence of these variables may introduce bias into the observed association between ADFI and PD risk, potentially distorting the true effect size. We must acknowledge our inability to collect and adjust for these specific factors; consequently, this limitation should be considered when interpreting the study findings. Additionally, although NHANES employs a sophisticated multistage stratified sampling design to select a representative sample, some degree of selection bias is inevitable, potentially affecting the generalizability of the findings. Finally, cases of PD in this study were defined on the basis of participants’ self‐reported use of anti‐Parkinsonian prescription medications identified within the NHANES dataset. However, this medication‐based definition carries inherent limitations and may introduce significant misclassification bias. Key limitations include: 1. The identified anti‐Parkinsonian medications (e.g., levodopa, dopamine agonists, MAO‐B inhibitors) lack specificity for PD, as they are also indicated for other movement disorders (such as essential tremor [ET] and restless legs syndrome [RLS]) or non‐movement disorder conditions. 2. Self‐reported medication use is susceptible to recall bias and reporting inaccuracies 3. This approach fails to identify untreated PD patients. These biases, particularly the potential for false positives, may attenuate the observed association between ADFI and PD toward the null, as the erroneous inclusion of non‐PD medication users in the case group could dilute genuine associations. Nevertheless, the significant associations observed may still indicate the presence of true effects. Given the reliance on publicly available NHANES data, the study protocol could not be further optimized to incorporate more definitive diagnostic criteria (such as neurological specialist evaluation, standardized diagnostic criteria, or neuroimaging biomarkers). Consequently, the medication‐based proxy definition represented the optimal approach available within the constraints of the NHANES data structure, yet the resulting misclassification bias constitutes a significant limitation of this study.

## 5 Conclusion

In summary, our study revealed a negative correlation between ADFI and PD risk. Compared with individuals with higher ADFI, those with lower ADFI had 68% higher odds of PD. Additionally, higher ADFI (per unit) was associated with 16% lower odds of PD. Therefore, individuals with lower ADFI intake may warrant prioritized attention regarding PD risk. The findings of this study provide some insight into the role of ADFI in the pathogenesis of PD and underscore the importance of a balanced diet, particularly emphasizing adequate DF intake. Additionally, there is a need for further longitudinal cohort studies to explore the deeper mechanisms and potential applications of the relationship between DF and PD.

## Author Contributions

**Qifan Yang**: conceptualization, methodology, software, validation, formal analysis, visualization, writing – original draft. **Shijin Li**: formal analysis, methodology. **Xiao Li**: methodology, formal analysis. **Jiayu Peng**: data curation, investigation. **Bowen Han**: data curation, investigation. **Yuetong Liu**: data curation, investigation. **Xuehui Chang**: project administration, resources, supervision, funding acquisition, writing – review and editing.

## Ethics Statement

The data used in this study are all from NHANES, which was conducted by the NCHS with appropriate ethical approval. Research involving human participants was reviewed and approved by the NCHS and the institutional Ethics Review Board (ERB). In accordance with national legislation and institutional requirements, written informed consent was not required for this study.

## Consent

The authors have nothing to report.

## Conflicts of Interest

The authors declare no conflicts of interest.

## Declaration of Generative AI and AI‐Assisted Technologies in the Writing Process

We confirm that no generative artificial intelligence (Generative AI) or AI‐assisted technologies were used in the writing process of this manuscript. All content was produced solely by the authors without any assistance from AI tools.

## Peer Review

The peer review history for this article is available at https://publons.com/publon/10.1002/brb3.70819

## Supporting information




**Supporting Material**: brb370819‐sup‐0001‐SuppMat.docx

**Supporting Table**: brb370819‐sup‐0002‐TableS1.xlsx

**Supporting Table**: brb370819‐sup‐0003‐TableS2.xlsx

**Supporting Table**: brb370819‐sup‐0004‐TableS3.xlsx

**Supporting Table**: brb370819‐sup‐0005‐TableS4.xlsx

**Supporting Table**: brb370819‐sup‐0006‐TableS5.xlsx

## Data Availability

This study analyzed data from an open‐access database. The original data for this study can be found at: https://wwwn.cdc.gov/nchs/nhanes/.
