# Supplementary material for: Association Between Dietary Fiber and Parkinson's Disease in the United States: NHANES 2005–2018
Source: Brain Behav. 2025 Sep 16;15(9):e70819. doi: 10.1002/brb3.70819 (PMC12441003; doi:10.1002/brb3.70819)
Supplement: Supplementary file 1 — Supporting Material: brb370819‐sup‐0001‐SuppMat.docx [file BRB3-15-e70819-s004.docx]

Anti-Parkinsonian Medication

| **NHANES Code** | **Drug** |
| --- | --- |
| **d00184** | Carbidopa |
| **d04877** | Carbidopa; Entacapone; Levodopa |
| **d03473** | Carbidopa; Levodopa |
| **d04537** | Rivastigmine |
| **d00086** | Amantadine |
| **h00026** | Acetaminophen; Amantadine; Chlorpheniramine |
| **h00031** | Acetaminophen; Amantadine; Chlorpheniramine;  Phenylephrine |
| **d04145** | Pramipexole |
| **d05848** | Rotigotine |
| **d04215** | Ropinirole |
| **d00976** | Selegiline |
| **d04991** | Apomorphine |
| **d00178** | Bromocriptine |
| **d04460** | Entacapone |
| **d00277** | Levodopa |
| **d04750** | Galantamine |
| **d04220** | Quetiapine |
| **d05612** | Rasagiline |
